# Supplementary material for: Copper Dysregulation in Major Depression: A Systematic Review and Meta-Analytic Evidence for a Putative Trait Marker
Source: Int J Mol Sci. 2025 Sep 22;26(18):9247. doi: 10.3390/ijms26189247 (PMC12470547; doi:10.3390/ijms26189247)
Supplement: Supplementary file 1 [file ijms-26-09247-s001.zip › ijms-3833424-supplementary.pdf]

PRISMA 2020 Main Checklist

Topic	No.	Item	Location where item is reported
**TITLE**			
**Title**	1	Identify the report as a systematic review.	Yes
**ABSTRACT**			
**Abstract**	2	See the PRISMA 2020 for Abstracts checklist	No
**INTRODUCTION**			
**Rationale**	3	Describe the rationale for the review in the context of existing knowledge.	Section 1, Page 2-3
**Objectives**	4	Provide an explicit statement of the objective(s) or question(s) the review addresses.	Page 3, Lines 103-109
**METHODS**			
**Eligibility criteria**	5	Specify the inclusion and exclusion criteria for the review and how studies were grouped for the syntheses.	Page 3, Lines 117-123
**Information sources**	6	Specify all databases, registers, websites, organisations, reference lists and other sources searched or consulted to identify studies. Specify the date when each source was last searched or consulted.	Page 3, Lines 126-137
**Search strategy**	7	Present the full search strategies for all databases, registers and websites, including any filters and limits used.	Page 4, Lines 140-143
**Selection process**	8	Specify the methods used to decide whether a study met the inclusion criteria of the review, including how many reviewers screened each record and each report retrieved, whether they worked independently, and if applicable, details of automation tools used in the process.	Page 4, Lines 144-148
**Data collection process**	9	Specify the methods used to collect data from reports, including how many reviewers collected data from each report, whether they worked independently, any processes for obtaining or confirming data from study investigators, and if applicable, details of automation tools used in the process.	Page 4, Lines 151-155
**Data items**	10a	List and define all outcomes for which data were sought. Specify whether all results that were compatible with each outcome domain in each study were sought (e.g. for all measures, time points, analyses), and if not, the methods used to decide which results to collect.	Page 4, Lines 156-166
	10b	List and define all other variables for which data were sought (e.g. participant and intervention characteristics, funding sources). Describe any assumptions made about any missing or unclear information.	Page 4, Lines 144-145 and 161-166

Topic	No.	Item	Location where item is reported
**Study risk of bias assessment**	11	Specify the methods used to assess risk of bias in the included studies, including details of the tool(s) used, how many reviewers assessed each study and whether they worked independently, and if applicable, details of automation tools used in the process.	Page 4, Lines 169-181
**Effect measures**	12	Specify for each outcome the effect measure(s) (e.g. risk ratio, mean difference) used in the synthesis or presentation of results.	Page 4, Lines 184-188
**Synthesis methods**	13a	Describe the processes used to decide which studies were eligible for each synthesis (e.g. tabulating the study intervention characteristics and comparing against the planned groups for each synthesis (item 5)).	Pages 5, Lines 191-193
	13b	Describe any methods required to prepare the data for presentation or synthesis, such as handling of missing summary statistics, or data conversions.	Page 4, Lines 161-166
	13c	Describe any methods used to tabulate or visually display results of individual studies and syntheses.	Pages 5, Lines 191-193
	13d	Describe any methods used to synthesize results and provide a rationale for the choice(s). If meta-analysis was performed, describe the model(s), method(s) to identify the presence and extent of statistical heterogeneity, and software package(s) used.	Pages 5, Lines 191-205
	13e	Describe any methods used to explore possible causes of heterogeneity among study results (e.g. subgroup analysis, meta-regression).	Pages 5, Lines 194-203
	13f	Describe any sensitivity analyses conducted to assess robustness of the synthesized results.	Pages 5, Lines 208-211
**Reporting bias assessment**	14	Describe any methods used to assess risk of bias due to missing results in a synthesis (arising from reporting biases).	Pages 5, Lines 214-219
**Certainty assessment**	15	Describe any methods used to assess certainty (or confidence) in the body of evidence for an outcome.	Page 4, Line 180
**RESULTS**			
**Study selection**	16a	Describe the results of the search and selection process, from the number of records identified in the search to the number of studies included in the review, ideally using a flow diagram.	Page 5, Lines 222-227
	16b	Cite studies that might appear to meet the inclusion criteria, but which were excluded, and explain why they were excluded.	Page 6, Figure 1
**Study characteristics**	17	Cite each included study and present its characteristics.	Pages 6-7, Table 1
**Risk of bias in studies**	18	Present assessments of risk of bias for each included study.	Pages 8-9, Table 2
**Results of individual studies**	19	For all outcomes, present, for each study: (a) summary statistics for each group (where appropriate) and (b) an effect estimate and its precision (e.g. confidence/credible interval), ideally using structured tables or plots.	Page 8, Figure 2

Topic	No.	Item	Location where item is reported
**Results of syntheses**	20a	For each synthesis, briefly summarise the characteristics and risk of bias among contributing studies.	Page 10, Figure 3
	20b	Present results of all statistical syntheses conducted. If meta-analysis was done, present for each the summary estimate and its precision (e.g. confidence/credible interval) and measures of statistical heterogeneity. If comparing groups, describe the direction of the effect.	Page 7, Lines 261-266
	20c	Present results of all investigations of possible causes of heterogeneity among study results.	Pages 9-10, Lines 299-322
	20d	Present results of all sensitivity analyses conducted to assess the robustness of the synthesized results.	Page 9, Lines 287-293
**Reporting biases**	21	Present assessments of risk of bias due to missing results (arising from reporting biases) for each synthesis assessed.	Page 10, Lines 325-334
**Certainty of evidence**	22	Present assessments of certainty (or confidence) in the body of evidence for each outcome assessed.	Page 7, Lines 261-236
**DISCUSSION**			
**Discussion**	23a	Provide a general interpretation of the results in the context of other evidence.	Page 11, Lines 353-382
	23b	Discuss any limitations of the evidence included in the review.	Page 12, Lines 408-412
	23c	Discuss any limitations of the review processes used.	Page 12, Lines 420-423
	23d	Discuss implications of the results for practice, policy, and future research.	Page 12, Lines 426-432; Page 13, Lines 443-445
**OTHER INFORMATION**			
**Registration and protocol**	24a	Provide registration information for the review, including register name and registration number, or state that the review was not registered.	review was not registered
	24b	Indicate where the review protocol can be accessed, or state that a protocol was not prepared.	review was not registered
	24c	Describe and explain any amendments to information provided at registration or in the protocol.	review was not registered
**Support**	25	Describe sources of financial or non-financial support for the review, and the role of the funders or sponsors in the review.	Page 13, Line 453
**Competing interests**	26	Declare any competing interests of review authors.	Page 13, Lines 454-455

Topic	No.	Item	Location where item is reported
**Availability of data, code and other materials**	27	Report which of the following are publicly available and where they can be found: template data collection forms; data extracted from included studies; data used for all analyses; analytic code; any other materials used in the review.	review was not registered

From: Page MJ, McKenzie JE, Bossuyt PM, Boutron I, Hoffmann TC, Mulrow CD, et al. The PRISMA 2020 statement: an updated guideline for reporting systematic reviews. MetaArXiv. 2020, September 14. DOI: 10.31222/osf.io/v7gm2. For more information, visit: www.prisma-statement.org
